# Media Forensic Considerations of the Usage of Artificial Intelligence Using the Example of DeepFake Detection

**DOI:** 10.3390/jimaging10020046

**Published:** 2024-02-09

**Authors:** Dennis Siegel, Christian Kraetzer, Stefan Seidlitz, Jana Dittmann

**Affiliations:** Department of Computer Science, Otto-von-Guericke University, 39106 Magdeburg, Germany; christian.kraetzer@ovgu.de (C.K.); stefan.seidlitz@ovgu.de (S.S.);

**Keywords:** artificial intelligence (AI), DeepFake, DeepFake detection, forensics, explainable AI (xAI), Artificial Intelligence Act (AIA), human–AI interfaces, system causability scale (SCS)

## Abstract

In recent discussions in the European Parliament, the need for regulations for so-called high-risk artificial intelligence (AI) systems was identified, which are currently codified in the upcoming EU Artificial Intelligence Act (AIA) and approved by the European Parliament. The AIA is the first document to be turned into European Law. This initiative focuses on turning AI systems in decision support systems (human-in-the-loop and human-in-command), where the human operator remains in control of the system. While this supposedly solves accountability issues, it includes, on one hand, the necessary human–computer interaction as a potential new source of errors; on the other hand, it is potentially a very effective approach for decision interpretation and verification. This paper discusses the necessary requirements for high-risk AI systems once the AIA comes into force. Particular attention is paid to the opportunities and limitations that result from the decision support system and increasing the explainability of the system. This is illustrated using the example of the media forensic task of DeepFake detection.

## 1. Introduction

The EU Artificial Intelligence Act (AIA) is a recent regulation for the use of artificial intelligence (AI) systems [1]. It utilizes a risk-based approach to regulate AI and formulate requirements specific to the individual risk level identified. These risk levels are unacceptable, high, limited, and minimal or no risk. The requirements range from transparency obligations at the lower end of the scale to prohibition. The key component of the requirements relates to the category of high-risk AI. This is not the first document to set requirements for the use of AI systems, but it is the first major one to be turned into European law. With the European Parliament’s recent amendments to the AIA [2], the detection of DeepFakes (as one specific form of AI application) is no longer directly classified as a high-risk application. This is a significant change from drafts of the AIA, which considered DeepFakes to be a greater risk to society. Nevertheless, even with the current state of the AIA, there are two specific contexts in which DeepFake detection would still be strongly regulated. These two contexts are AI usage (including DeepFake detection) in law enforcement (see [1,2], Annex III, 6) and AI usage in Biometric Identification (see [1,2], Annex III, 1).

In general, the AIA foresees human oversight and control as strict requirements for high-risk AI application contexts and systems. This will have significant impact on AI system designs and implementations in order to keep the human ‘in the loop’, as is required by this regulative act. As an additional consequence, the integration of the human factor will also have an impact on the error behavior of such future AI systems. The efficiency of the communication of AI results to the operator in decision support systems will significantly support or thwart the operators’ reactions and thereby (among other factors) the resulting overall decision accuracy. While a tremendous amount of research has been invested in the recent past into AI methods and the evaluation of their decision performance, only a very limited amount of effort has, in the same period of time, investigated efficient AI-to-operator communication and the corresponding performance evaluations. In this paper an existing evaluation scheme to subjectively evaluate the quality of the human–computer interface (HCI); this interface is used as explanation interface, or an explanation process called the System Causability Scale (SCS) is extended to better fit the requirements of an exemplary AI system application scenario. The scenario used here for illustration is the AI application domain of DeepFake detection. DeepFakes, as a recent trend in media manipulations that replace authenticity- and identity-related information (faces, voices, and also spoken content) in video files or streams, are a significant threat to modern society and any assumption of trust in image, video, and audio content. With an adapted and enhanced set of qualitative methods aiming to subjectively assess requirements for high-risk AI systems, as introduced in this paper, operators (who are usually experts in their tasks and are therefore valuable sources for feedback and the individuals most likely to notice abnormal/unfair AI behavior in their expert domain) can and should be included in the design and evaluation of such AI systems.

In this paper, the steps required for the usage of AI application in the forensic context are partially addressed by the following contributions:

- A literature review of regulatory documents on the usage of AI technology;
- Identification of 15 requirements for the use of high-risk AI systems and their implications in the context of DeepFake detection;
- Discussion of the possibilities and challenges of explainability in AI applications, taking into account different audiences of the explanations.

This paper is structured as follows: Section 2 provides a overview on the relevant aspects of DeepFakes and existing proposals for the regulation of AI, with particular emphasis on forensics and the explainability in AI systems. In Section 3, the identified requirements for the usage of AI are presented. Section 4 applies the previously discussed requirements to the context of DeepFake detection. At the end of the paper, in Section 5, a discussion of the authors’ perspective is presented.

## 2. The State of the Art

With regard to the use of DeepFake detection in a forensic context, with the aim of using it in court, various basic principles are required. First, a brief introduction to the topic of DeepFakes is given in Section 2.1. Afterwards, existing and upcoming regulations for the usage of AI are discussed in Section 2.2. Explainability, as one of the identified requirements, will then be discussed in more detail in Section 2.3.

### 2.1. DeepFakes

DeepFakes are a current threat to modern society, significantly impairing trust in digital image, video, and audio content due to the possibility of replacing identity-related information (faces, voices, and also spoken content) in media files or streams. DeepFake detection as a countermeasure to this threat is an important measure, and since 2017, a very fast-growing field of research that has easily exceeded the 1000 publications mark within the last six years. Presenting an overview of the domain of AI and decision support systems is outside of the scope of this paper. An overview of the field can be found in [3]. The authors’ own research work in DeepFake detection is illustrated, e.g., in [4,5].

### 2.2. Regulatory Requirements for the Usage of AI

With the possibility of manipulating digital media using DeepFake technology, authenticity verification is becoming even more important. This applies in particular to use in court in the context of media forensics. In the U.S., the Federal Rules of Evidence (FRE [6], FRE 702 [7]) and the Daubert criteria in particular, set out the necessary requirements for the admission of evidence in court. Potential usage in Europe is also discussed in [8]. An extended discussion on the Daubert criteria and FRE702 can be found in [4]. For the authors of this paper, the German situation is relevant.The German Federal Office for Information Security (BSI) has provided guidelines for IT forensics. Currently, the most relevant guideline is the “Leitfaden IT-Forensik” [9]. The so-called Data-Centric Examination Approach (DCEA) [10] builds on this. The DCEA consists of three key aspects, which are a phase-driven process model and categorization of forensic data types and method classes. Said phase model and forensic data types are used and specified for the context of media forensics and DeepFake detection in [5]. As far as method classes are concerned, the European Network of Forensic Science Institutes (ENFSI) also provides various best-practice documents, including one for image authentication methods [11]. One such best-practice document is used in [12] and specified for the context of DeepFake detection.

However, these documents do not focus on or specify the usage of AI. This will become necessary when the EU Artificial Intelligence Act (AIA) comes into force in the future. At the time of writing, the AIA has been proposed by the European Commission [1] and approved with slight changes by the European Parliament [2]. In the AIA, a categorization of AI systems based on risk levels is performed. In the initial proposal DeepFakes (including their detection) were classified as high-risk; however, in further discussions resulting in the current iteration of the document [2], DeepFake detection no longer falls into the high-risk category in general, but depends on specific circumstances. One of these circumstances is the usage of AI in the context of Biometric Identification (see [1], Annex III, 1). Taking into account the report of ENISA regarding remote identity proofing [13], DeepFakes may become a challenge in this scenario. With regard to risk levels, low-risk applications only have transparency obligations. As the risk classification increases in severity, so do the requirements. A more detailed description of requirements for high-risk applications can be found in Section 3.

In addition, various documents regarding the usage of AI systems have been proposed in recent years. Different documents use different terminology for specific aims i.e., “Trustworthy AI” [14,15,16], “Responsible AI” [17] or “Auditable AI” [18,19]. The BSI also provides a guideline for the auditing of AI systems [19]. With regards to individual requirements of AI they refer to AI quality criteria defined by the German Institute for Standardisation Registered Association (DIN) in [18]. In their own words, they focus on the ethical aspects of AI usage and define a total of nine criteria. Among other things, the role of humans in the process (both users and affected parties) and external influences such as the General Data Protection Regulation (GDPR) [20] are discussed. Another recommendation for the usage of AI comes from the United Nations Educational, Scientific and Cultural Organization (UNESCO). It also focuses on the ethical aspects and places particular emphasis on the human being in and around the process. The unique aspects of this document are public awareness of AI, sustainability of AI application, and broad definitions of transparency and explainability. In “Four Principles of Explainable Artificial Intelligence” by the National Institute of Standards and Technologies (NIST) [16], two aspects are addressed: requirements for trustworthy AI systems with a focus on explainable AI, and four principles of explainable AI. The latter is more relevant and will be discussed in more detail in the following Section 2.3. The last and most recent document addressing responsible AI originates from Interpol [17]. It also contains the most important requirements of the documents discussed. A overview of the individual requirements will be described in Section 3.2.

One specific aspect of the AI lifecycle is also addressed in [21], which refers to the training of DeepFake detection algorithms. Here, the requirements of certification and the benchmarking of these algorithms are identified.

### 2.3. Explainability in Artificial Intelligence

Although the aspect of explainability is just one of the requested requirements for the usage of artificial intelligence, it must be particularly emphasized. This is due to the fact that in the literature, there are differences in the terminology and criteria for achieving explainability. In research, this term is often referred to as “Explainable AI”, or XAI for short. Interpol defines explainability “[…] *aims to ensure that even when humans cannot understand ‘how’ an AI system has reached an output, they can at least understand ‘why’ it has produced that specific output. This field distinguishes explainability in a narrow sense, as different from interpretability.”* [17]. So in other words, the described aspect of explainability is hoped to provide the reasoning for a particular outcome of an AI system, which is also called local explanation [22]. In contrast, the BSI focuses on the underlying model used to reach that decision [19]. Explaining the model requires knowledge on the specific architecture used and its parameterization, also called global explanation [22]. In addition, information regarding the training data is necessary, which in the context of DeepFake detection may further be influenced by data protection regulations.

A different aspect of explainability is focusing on the explanations themselves. The system causability scale (SCS), proposed by Holzinger et al., is used to measure explanations based on a scoring system [23]. It consists of ten questions for quality estimation of explanations of AI systems. From the user answers, a rating scale is calculated using a Likert scale ([24]). The final score is an indicator of how explainable the AI system is from the users’ perspective. An opposing view is given by Rosenfeld et al., stating subjective evaluation to be unreliable due to potential confirmation bias [25]. Instead, they focus on four different metrics for an objective measurement. These essentially quantify the potential performance loss due to a more transparent model, as well as the complexity and stability of the explanation provided and formulated in a mathematical equation. NIST [16] also focuses on the explanation by identifying four principles of explainable AI. These are “Explanation”, “Meaningful”, “Explanation Accuracy” and “Knowledge Limits”. The latter three are the explanation properties which should be fulfilled for human interaction. The aspect of “Meaningful” further introduces different recipients of the explanation with different knowledge and experience. For the forensic context of DeepFake detection, the roles of data scientist, forensic examiner, and person affected by the AI were identified in [12]. However, these roles are context-dependent and might differ or have additional roles in other contexts. The second property of “Explanation Accuracy” focuses on the correctness of the given explanation with regard to the actual decision-making process of the AI system. It has to be noted that “Explanation Accuracy” is distinct from the detection performance and solely focuses on the specific explanation. In addition, NIST proposes different “styles” of explanations, based on the interactiveness of the explanation with the human operator. “Knowledge Limits” focuses on the boundary conditions of the systems and provides background information on when the system should not be used (i.e., by giving reasoning for low-confidence decisions. In [26], an instance of ChatGPT is trained to interpret and explain potential cybersecurity risks, which can further be explained by the interaction of the user.

With regard to the types of explanation, NIST differentiates between self-interpretable models and post-hoc explanations. Self-interpretable models refer to methods of traditional machine learning, such as decision trees and rule-based decision making. This group of methods is also referred to as glass or white box [18], “shallow AI”, or symbolic AI [19]. In contrast, post-hoc explanations are most commonly used for deep learning-based algorithms, which are often referred to as black box or connectionist AI [19]. For said post-hoc approaches, most often, some form of heatmap is used to increase explainability in DeepFake detection (i.e., LRP [27], LIME [28], SHAP [29]), or GRAD-CAM [30]. In addition, there are specific explanation tools tied to specific classification algorithms (for example to random forests [31]). Rudin et al. [32] further discuss the use of post hoc approaches in general and whether they can be replaced by more transparent algorithms.

## 3. Derived Requirements for the Context of DeepFake Detection

Based on the various documents mentioned in Section 2.2, we have identified a total of 15 requirements which will be relevant once the EU AIA comes into force. These requirements will be described in Section 3.1 based on the definitions given in the EU AIA and related sources and expanded upon when necessary. The applicability of the requirements is then validated by projecting the existing documents onto the requirements in Section 3.2.

### 3.1. Selected Definitions from the Context of the AIA

As mentioned previously, under particular circumstances, DeepFake Detection will also fall in the category of high-risk AI. Because of that, initial focus on individual requirements is placed on the EU AIA (see [1,2]). The EU AIA itself contains no definitions but relies strongly on well-defined terminology that has, in advance of the drafting process, been established by a so-called High-Level Expert Group on AI (HLEG). In the AIA, the corresponding process (which is outlined in ([33]) is summarized as follows: “*The proposal builds on two years of analysis and close involvement of stakeholders*, […]. *The preparatory work started in 2018 with* […] *52 well-known experts tasked to advise the Commission on the implementation of the Commission’s Strategy on Artificial Intelligence. In April 2019, the Commission supported* […] *the key requirements set out in the HLEG ethics guidelines for Trustworthy AI*” ([34]) The Assessment List for Trustworthy Artificial Intelligence (ALTAI; [14]) published those requirements in 2020.

From the definitions given in the ALTAI and their usage in the AIA [1,2], the following terminology is derived here to enable the presentation of a categorization of requirements for high-risk AI systems in Section 3.2. For the exemplary selected application domain of DeepFake detection, this is then used in Section 4 for introducing a projection of qualitative methods aiming to assess those requirements for which the evaluation would benefit from including subjective expert operator opinions. This pays respect to the fact that the efficiency of the communication of AI results to the operator in decision support systems will significantly assist or thwart the operators reactions, and thereby will also affect (among other factors) the resulting overall decision accuracy.

In total, we identified 15 requirements, which are presented in Table 1. These 15 requirements can be broken down into two groups. The first group of 12 requirements in the list should always be considered. However, the last three criteria, namely Legal Framework Conditions, Human, Social & Environmental Well-being, and Ethical and normative Guidelines (incl. Human-centered Values) are context-dependent (i.e., governed by national legislation) and thus might be different or change over time. In addition to the brief description of the individual requirements, a more detailed explanation will be given in the following subsections.

#### 3.1.1. Data Protection (incl. Privacy Protection)

AI systems should respect and uphold privacy rights and corresponding data protection regulations and ensure the confidentiality, integrity, authenticity, and non-repudiation of the data. This includes training, validation, and testing data sets containing person-related information. The ALTAI specifies aspects regarding the Data Protection Impact Assessment (DPIA) as well as the role of the Data Protection Officer (DPO).

#### 3.1.2. Reliability, Safety, and Run-Time Constraints

AI systems should reliably operate in accordance with their intended purpose throughout their life cycle. The defined run-time constrains should be kept accordingly, even under potentially significantly growing workloads. This is also the main intention of the ALTAI, which addresses reliability with AI reliability.

#### 3.1.3. Accountability, Autonomy, and Control

The ALTAI ([14]) defines accountability as follows: “*This term refers to the idea that one is responsible for their action—and as a corollary their consequences—and must be able to explain their aims, motivations, and reasons.* […] *Accountability is sometimes required by law. For example, the General Data Protection Regulation (GDPR) requires organisations that process personal data to ensure security measures are in place to prevent data breaches and report if these fail. But accountability might also express an ethical standard, and fall short of legal consequences.*” Regarding the control of AI, the AIA ([1]) strongly requires human oversight, which is justified in the ALTAI ([14]) as follows: “*Human oversight helps ensure that an AI system does not undermine human autonomy or causes other adverse effects. Oversight may be achieved through governance mechanisms such as a human-in-the-loop (HITL), human-on-the-loop (HOTL), or human-in-command (HIC) approach*.” As a consequence of human oversight, those responsible for the various phases of the AI system life cycle should be identifiable and accountable for the outcomes of the system.

#### 3.1.4. Transparency of Algorithms

The design of a decision algorithm of an AI system and the criteria and results of its evaluation should be made public (either to the general public or for a specific audience (e.g., certified auditors)). The ALTAI is clear that the transparency of an algorithm does not necessarily “*imply that information about business models and Intellectual Property related to the AI system must always be openly available*”. The ALTAI considers this criterion to be a part of algorithm auditability and explainability.

#### 3.1.5. Algorithm Auditability and Explainability

There should exist methods that enable third parties to examine and review the implementation and behavior of an algorithm and thereby allow for the identification of what the AI system is doing and why. This may include detailed descriptions of the system’s architecture, processes and their implementation, trained models (incl. training data), and input data. This definition is similar to the definition of Auditability in ALTAI, except for the fact that the conceptual design of a decision algorithm was shifted into a separate criterion.

#### 3.1.6. Usability, User Interface Design and Fairness (Accessibility)

The user interface design for an AI system should enable understandability of decisions and explanations. AI systems should be inclusive and accessible (independent of disabilities) in usage and should not result in discrimination against individuals, communities, or groups. Those criteria are addressed in detail in ALTAI, under Universal Design.

#### 3.1.7. Accuracy, Decision Confidence, and Reproduceability

AI systems must have a known (or potential) and acceptable error rate. For each decision, a level decision confidence in its result should be communicated. In addition, the AI system must come to the same decision and confidence with the same input. The ALTAI specification of Accuracy places the generalization of unseen data in AI systems in the foreground. They define Accuracy as “*the fraction of predictions the model got right*’’ ([14]). Further, ALTAI defines the decision confidence using the Confidence Score.

#### 3.1.8. Fairness (Non-Biased Decisions)

Results of AI system usage (i.e., decisions and their confidence) should not involve or result in unfair discrimination against individuals, communities, or groups. ALTAI describes Fairness in more detail, but the key message is the same.

#### 3.1.9. Decision Interpretability and Explainability

Every decision made by the AI system must be interpretable and explainable, together with an confidence estimate for this decision. This definition is coherent with the definition of Interpretability in ALTAI and does not necessarily imply that everyone can make this interpretation. It might require very specific knowledge of training.

#### 3.1.10. Transparency and Contestability of Decisions

There should be transparency and responsible disclosure so that people know when they are significantly impacted by an AI system and can find out when an AI system is engaging with them. When an AI system significantly impacts a person, community, group or environment, there should be a timely process to allow people to challenge the use or output of the system.

ALTAI specifies Transparency in Requirement #4, which is sub-divided into the three elements: Traceability, Explainability and Open Communication. Traceability addresses the “[*a*]*bility to track the journey of a data input through all stages of sampling, labelling, processing and decision making*”. In contrast, Explainability refers to the “*technical processes of the AI system and the reasoning behind the decisions or predictions that the AI system makes*”. The third category, Open Communication, relates to the capabilities and limitations of the AI system which have to be communicated to users.

#### 3.1.11. Legal Framework Conditions

The usage of AI systems is governed by (inter)national legislation. For each AI application, a corresponding legal situation has to be accessed and considered in the design, implementation, configuration and operation of the system. Adherence to these requirements has to be documented. These aspects are addressed in HITL, HOTL and HIC of the ALTAI specifications, where HIC is referred to as “*the capability to oversee the overall activity of the AI system* (*including its broader economic, societal, legal and ethical impact*) *and the ability to decide when and how to use the AI system in any particular situation. The latter can include the decision not to use an AI system in a particular situation to establish levels of human discretion during the use of the system, or to ensure the ability to override a decision made by an AI system*”.

### 3.2. Projection of the Derived Requirements in the Existing Literature

**Figure 1:**
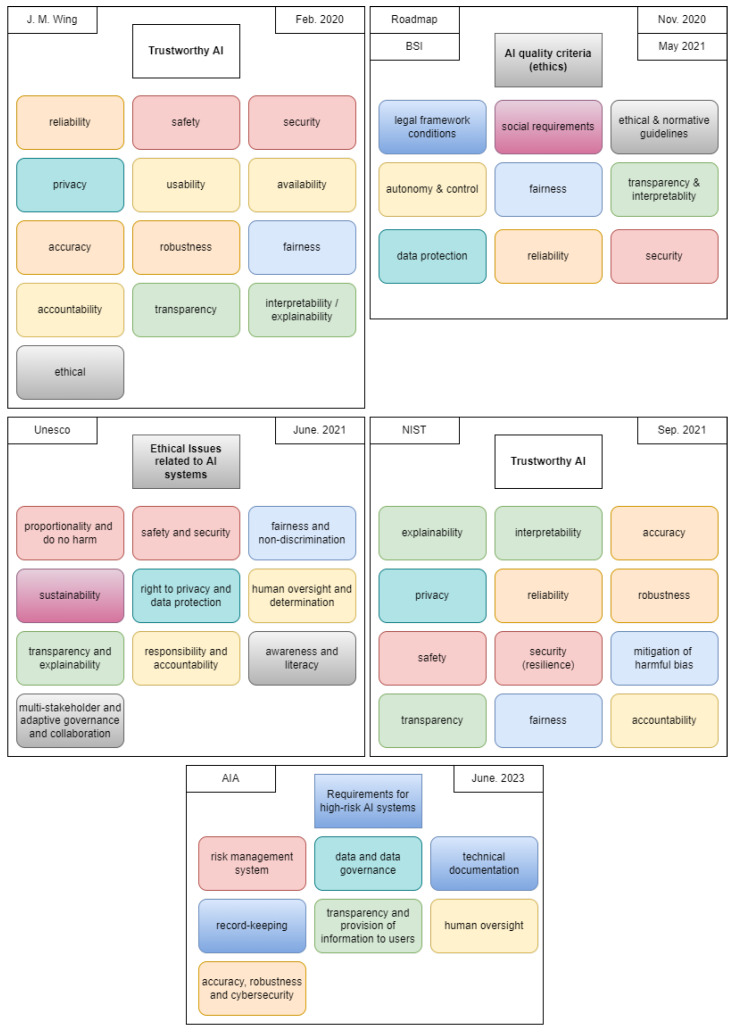
Identification of requirements in the existing literature for the references [1,2,15,16,18,19,36]. Identical colors for individual blocks indicate a similar or identical scope of the requirement.

**Figure 2:**
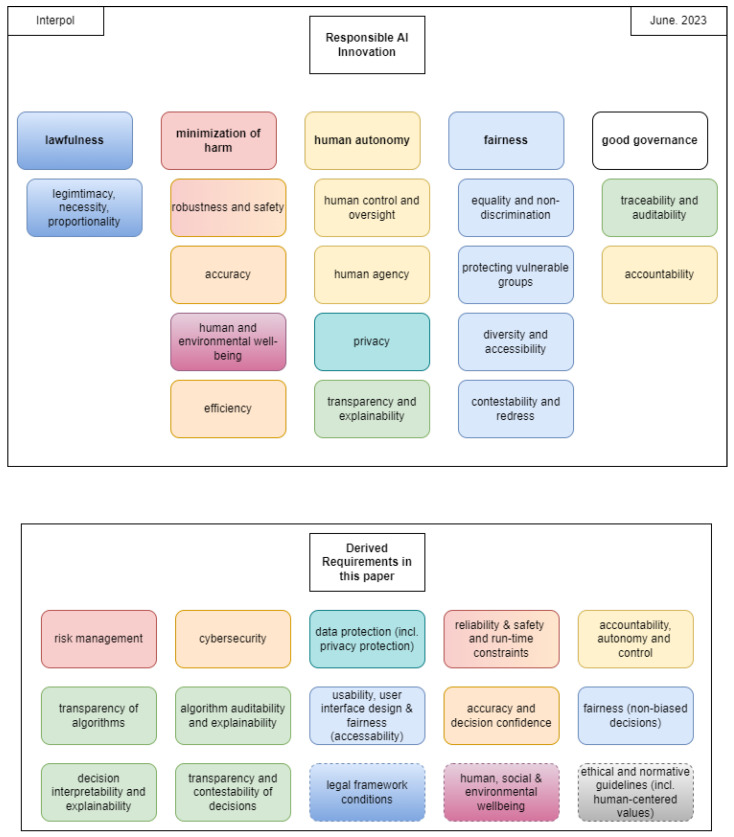
Continuation of Figure 1 with the addition of [17] and the derived requirements in this paper. Requirements in dotted lines indicate external influences that potentially overlap and might be addressed by other requirements.

To further validate the suitability of the derived requirements, a projection is performed with regard to existing documents addressing the usage of AI systems, as discussed in Section 2.2. However, it has to be noted that the different documents have different terminology for the same definition, which reinforces the need for uniform terminology. In Figure 1 and Figure 2, the requirements of existing literature are shown and colored to highlight matching scopes and definitions. In contrast to the existing documents, we diversified the aspect of explainability to differentiate between the algorithm and its decisions as well as different recipients of the explanation, which are most commonly used ambiguously. A further illustration of the comparison can be found in Table 2. The first column of the mapping relates to the AIA, as this is the focus of the work. The order afterwards corresponds to the date of publication of the document, starting with the oldest on the left-hand side. In particular, it should be noted that the area of requirements is constantly evolving, as only some specific aspects were dealt with in the first documents. In addition, the requirements are categorized according to their scope of application, resulting in a total of five categories, as shown in the first column of Table 2. The first category, IT Systems and Protocols and Compliance, summarizes all aspects of the IT system and is not tied solely to AI systems. It addresses, among other things, the principles of the forensic process (i.e., the chain of custody and logging, as described in [5]). Algorithms and their training refers to the steps of algorithm and model development before they are applied. For more details on model development (i.e., forensic modeling work as well as benchmarking and certification), the reader is referred to [5,21,35]. UI Usability concerns the general application of the IT system’s user interface. The fourth category of Decisions refers to the application of the system in individual cases (e.g., DeepFake detection in remote identity proofing [13]). In this example, a distinction is made between the human operator of the AI system and the one affected by the decision. The last category consists of Legal Framework Conditions; Human, Social and Environmental Well-being; Ethical and Normative Guidelines (incl. Human-centered Values); and External Influences. As they are highly dependent on national legislation and norms, they are not considered in detail in this paper.

## 4. Challenges in AI Requirements for DeepFake Detection

With the aforementioned considerations of DeepFake detection being classified as high-risk, the requirements identified in Table 2 have to be addressed. Particular focus is placed on explainability and human-in-the-loop aspects, as these should be integrated into DeepFake detection and the associated detectors. According to the categorization of requirements, they correspond to Algorithms and their training, UI Usability and Decisions.

On the subject of DeepFake detection, this paper divides AI systems into strong and weak AI. Strong AI refers to classification algorithms without the necessity of human intervention or interaction. According to the current state of research, most approaches to DeepFake detection fall into this category. On the other hand, weak AI include humans to the decision making in AI systems, implementing the concept of human-in-the-loop. This requires different methods, such as interface for explainability, which can be classified as quantitative and qualitative methods. Quantitative methods cover explanations of the algorithm decision process (e.g., visualization or textual descriptions) in AI systems, which can be used by the user of an AI system. In qualitative methods, humans have a stronger influence in controlling the training and classification of AI systems. As shown by [37,38], the addition of human-in-the-loop can also be used for data quality assurance. However, the integration of a human overseer into AI systems could introduce new error sources into the overall decision. The best examples of this are confirmation bias and misinterpretations by the system user, which might have a negative impact on the AI system’s decision-making performance. Potential security and privacy issues with regards to HITL in the environment of the Internet of Things can be found in [39].

However, this aspect of HITL has to be considered in a differentiated manner, as different roles and actors are involved. As discussed in Section 2.3, the roles and actors involved in the usage of AI are always context-dependent. The following assumes a forensic investigation is performed with the aim of DeepFake detection for evidence collection and use in court. In this specific context, we identified a total of six different (potential) actors, all of them with different assumed technical backgrounds and therefore requiring different types of explanation. However, only four of these six are considered in more detail, namely the forensic expert, the person affected by the AI decision, the data scientist, and the actor representing the legal point of view. The other two are the social and the ethical point of view, which originate from external influences. In addition, it should be noted that forensic science is regulated by national legislation and the identified actors reflect these forensic standards in Europe and especially in Germany. The list of actors and the description of their roles could therefore differ or change in the future.

With regard to the categories mentioned in Table 2, the applicability can be mapped to individual actors. The category of IT Systems, Protocols and Compliance relates in particular to the legal point of view. Algorithms and their training are addressed by the data scientist. To address the requirements of Transparency of Algorithms and Algorithm Auditability and Explainability, more details on the algorithmic processinghave to be provided, e.g. by highlighting individual processing steps. For this purpose an atomic layout is presented in [5,21]. In addition, benchmarking procedures have to be established (including the management of data sets considered for training and testing), as discussed in [35]. UI Usability is not tied to a specific role and should ideally be suitable for everyone involved. Explainability with regards to Decisions refers to the application of the system in individual cases (e.g., DeepFake detection in remote identity proofing). This is primarily relevant to the roles of forensic examiner and the person affected by the AI. It aims to achieve local explainability, as described in Section 2.3. In a previous work, decision tree classifiers were integrated into a user interface to enable the visualization of all features considered for the classification and their influence on the decision [12]. However, this form of explanation is less suitable for deep learning-based approaches, as their feature spaces are much larger and more abstract. In addition, the proposed interface is tied to the role of the forensic examiner, and aspects such as the forensic methods and data types are less relevant to the person affected by the decision. For image-based classification, saliency maps are currently the most prominent way to highlight relevant areas images in neural network-based classification. Saliency maps can be created based on pixels (e.g. by LRP [27]), image parts (e.g. LIME by [28]), or image comparison (e.g. ProtoPNet by [40]). Gradient-weighted Class Activation Mapping (Grad-CAM [30]) appears to be one of the more suitable approaches, since it is also recommended by the BSI in [41]. We agree with the findings in [41] that not every local explanation approach is suitable for every model; non-reproducibility may also be experienced when using LIME [28] in default parametrization. In DeepFake detection, the saliency map can be used to validate the decision, as only some areas of a person should be considered relevant. To be more precise, this should highlight the specific regions relevant to individual detectors (e.g., the eye region for blinking detectors, as proposed in [12]). Finally, the category External Influences corresponds to the actor’s legal, social, and ethical point of view according to the corresponding requirements.

Qualitative methods are also used to check the quality of conformity with the identified criteria. In general, qualitative methods focus on the connection between the AI system and the system users. Based on the visualization given by quantitative methods, qualitative methods can be used to gather user feedback and improve the explainability of the AI part and its communication towards the user in a decision support system. This user feedback is based on carefully designed questions (e.g., the ones discussed in [42]) that must be answered both in text and through ratings. For that purpose, the so-called System Causability Scale (SCS) proposed by [23] contains ten questions for quality estimation of explanations of AI systems. From the user answers, a rating scale is calculated using a Likert scale ([24]). The final score is an indicator of how explainable the AI system is considered to be from a specific user’s perspective. In [12], a similar approach was taken. Questions linked to the individual features of a classifier are integrated in order to check the plausibility of the resulting decision. In accordance with the SCS, questions are designed and categorized to verify the compliance with the individual requirements. In addition to the existing ten questions of the SCS, five further questions are integrated. As shown in Table 3, these questions can be used in the identified requirements to validate the conformity based on the explanation provided. In contrast to the SCS, there is currently no rating score included, which would have to be added if this scheme were to be used in practice (which is outside of the scope of this paper). The questions are kept as generic as possible so that anyone can answer them, regardless of their role and experience. It should be noted that these questions only serve as a starting point and must be expanded and specified on the basis of the integrated DeepFake detectors.

**Figure 3:**
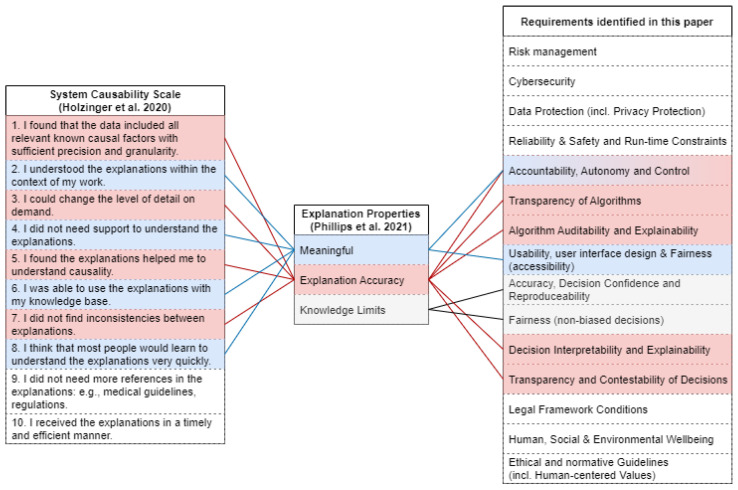
Illustration of properties of explanation by Phillips et al. [16] as addressed by SCS questions [23], and the applicability of SCS to the requirements identified in this paper.

A further connection can be made by the explanation properties provided in [16], as the aspects ”Meaningful”, “Explanation Accuracy”, and “Knowledge Limits” can be projected onto the existing questions of the SCS [23] as well as the requirements identified in this paper. An illustration of this connection can be found in Figure 3. However, these properties can only be considered in conjunction with a specific explanation.

## 5. Summary and Conclusions

Depending on the use case, DeepFake detection is performed either fully automated or with decision support systems. The latter should be the case in forensic application scenarios, where the results are intended to be used in court cases, making it a high-risk application of AI. In these use-cases, a highly trained expert (a forensic image or video analyst) has to rely on the efficiency of the communication of AI results to the operator in such decision support systems. The fact that these users are, per definition, domain experts makes them candidates very likely to notice abnormal/unfair AI behavior; thus, alongside fulfilling the ‘human oversight’ criterion, they are a valuable source of feedback foe evolving the AI and HCI components in the system. The integration of such qualitative feedback methods should be already included in AI system design and implementation processes. In operational systems, this aspect should be evaluated in terms of quality assurance and proficiency testing.

Alongside considerations of AI and HCI components, users of AI-driven decision support systems also need special training to efficiently implement ‘human oversight’ and to detect, interpret, and efficiently handle misclassifications caused by AI components.

It is a greater challenge to provide an explanation to the person affected by the AI decision that is “meaningful” according to NIST [16]. With the varying technical background knowledge of those involved, it is still unclear which levels of detail are necessary, especially for affected people without technical background, for understanding the explanation. Without that background knowledge, it may not even be possible to provide a meaningful explanation. Alternatively, existing algorithms can be validated using independent software for verification, so-called universal verification. Among other things, this can be used to investigate the learning behavior of generative AI. One possible question is how these methods can replicate their data basis. Considering the aspect of explainability, this is a necessary step to identify and explain the origin of potentially new sources of errors in DeepFake detection. This aspect will be explored in a future publication.

In summary, this paper aims to provide a perspective on the increasing relevance that the upcoming EU AIA will assign to the usage of AI applications and humans in the context of AI applications and the benefits this might have for decision explainability if considered early on in systems’ design and evaluation.

## Figures and Tables

**Table 1:** Overview of requirements for high-risk AI systems (descriptions based on [19], extended on basis of [16,17] and re-structured by the authors) in the context of the current version of the AIA [1,2].

Requirements	Descriptions
Risk management	The risk management system shall consist of a continuous iterative process run throughout the entire life cycle of a high-risk AI system, requiring regular systematic updating. It shall identify, estimate and evaluate current and arising risks.
Cybersecurity	High-risk AI systems shall be designed, implemented and configured in such a way that they achieve an appropriate level of cybersecurity (i.e., resilience against targeted attacks) and perform consistently in this respect throughout their lifecycle.
Data Protection (incl. Privacy Protection)	AI systems should respect and uphold privacy rights and corresponding data protection regulation and ensure the confidentiality, integrity, authenticity and non-repudiation of the data. This particularly includes training, validation and testing data sets containing person-related information. A more detailed description is given in Section 3.1.1.
Reliability and Safety and Run-time Constraints	AI systems should reliably operate in accordance with their intended purpose throughout their life cycle. The defined run-time constrains should be kept throughout the whole life cycle, even under potentially significantly growing workloads. A more detailed description is given in Section 3.1.2.
Accountability, Autonomy and Control	Those responsible for the various phases of the AI system life cycle should be identifiable and accountable for the outcomes of the system, and human oversight of AI systems should be enabled. A more detailed description is given in Section 3.1.3.
Transparency of Algorithms	The decision algorithm of the AI system and its evaluation criteria and results should be made public (either to the public or for a specific audience (e.g., certified auditors)). A more detailed description is given in Section 3.1.4.
Algorithm Auditability and Explainability	There should exist methods that enable third parties to examine and review the behavior of an algorithm and thereby allow them to identify what the AI system is doing and why. This may include detailed descriptions of the system’s architecture and processes, trained models (incl. the training data) and input data. A more detailed description is given in Section 3.1.5.
Usability, User Interface Design and Fairness (accessibility)	The user interface design for an AI system should enable understandability of decisions and explanations. AI systems should in their usage be inclusive and accessible (independent of disabilities), and should not involve or result in unfair discrimination against individuals, communities, or groups. A more detailed description is given in Section 3.1.6.
Accuracy, Decision Confidence and Reproduceability	AI systems must have a known (or potential) and acceptable error rate. For each decision, a level of decision confidence in its result should be communicated. A more detailed description is given in Section 3.1.7.
Fairness (non-biased decisions)	Results of AI system usage (i.e., decisions) should not involve or result in unfair discrimination against individuals, communities, or groups. A more detailed description is given in Section 3.1.8.
Decision Interpretability and Explainability	Every decision made by the AI system must be interpretable and explainable, together with an confidence estimate for this decision. A more detailed description is given in Section 3.1.9.
Transparency and Contestability of Decisions	There should be transparency and responsible disclosure to ensure people know when they are being significantly impacted by an AI system and can find out when an AI system is engaging with them. When an AI system significantly impacts a person, community, group, or environment, there should be a timely process to allow people to challenge the use or output of the system. A more detailed description is given in Section 3.1.10.
Legal Framework Conditions	The usage of AI systems is governed by national and international legislation. For each AI application, the corresponding legal situation has to be accessed and considered in the design, implementation, configuration, and operation of the system. Adherence to these requirements has to be documented. A more detailed description is given in Section 3.1.11.
Human, Social and Environmental Well-being	AI systems should benefit individuals, society, and the environment.
Ethical and Normative Guidelines (incl. human-centered values)	Amongst other issues, AI systems should respect human rights, diversity, and the autonomy of individuals.

**Table 2:** Categorization, projection and comparison of the requirements proposed in this paper with existing recommendations regarding the usage of AI systems.

Category	Requirements	AIA [1,2]	BSI [18,19]	Unesco [36]	NIST [16]	Interpol [17]
**IT Systems, Protocols and Compliance**	**Risk management**	Risk management system (Art. 9)	-	Proportionality and Do No Harm	-	-
	**Cybersecurity**	Accuracy, robustness and cybersecurity (Art. 15)	Security	-	Security (resilience)	-
	**Data Protection (incl. Privacy Protection)**	Data and data governance (Art. 10)	Data protection	Right to Privacy, and Data Protection	Privacy	Privacy
	**Reliability and Safety and Run-time Constraints**	-	Reliability; Safety	-	Reliability; Robustness; Safety	Robustness and Safety
	**Accountability, Autonomy and Control**	Human Oversight (Art. 14)	Autonomy and control	Responsibility and accountability	Accountability	Human autonomy (i.e., Human Control and Oversight, Human Agency) Accountability
**Algorithms and their training**	**Transparency of Algorithms**	Technical documentation (Art. 11)	Transparency and interpretability	-	Transparency	Transparency
	**Algorithm Auditability and Explainability**	Technical documentation (Art. 11)	-	-	Explainability	Traceability and Auditability
**UI Usability**	**Usability, User Interface Design and Fairness (accessibility)**	-	-	Fairness and non-discrimination	-	Fairness (i.e., Diversity and Accessibility)
**Decisions**	**Accuracy, Decision Confidence and Reproduceability**	Accuracy, robustness and cybersecurity (Art. 15)	-	-	Accuracy	Accuracy
	**Fairness (non-biased decisions)**	Data and data governance (Art. 10)	Fairness	Fairness and non-discrimination	Fairness; Mitigation of harmful bias	Fairness (i.e., Equality and Non-discrimination, Protecting Vulnerable Groups)
	**Decision Interpretability and Explainability**	Human Oversight (Art. 14)	-	Human oversight and determination;Transparency and explainability	Explainability; Interpretability	Explainability
	**Transparency and Contestability of Decisions**	Transparency and provision of information to users (Art. 13)	-	Transparency and explainability	Transparency	Fairness (i.e., Contestability and Redress)
**External Influences**	**Legal Framework Conditions**	The document itself states legal conditions for EU. Technical documentation (Art. 11) and record keeping (Art. 12)	Legal framework conditions	-	-	Lawfulness (i.e., Legitimacy, Necessity, Proportionality)
	**Human, Social and Environmental Well-being**	-	Social requirements	Sustainability; Awareness and literacy	-	Minimization of Harm (i.e., Human and Environmental Well-being, Efficiency)
	**Ethical and Normative Guidelines (incl. Human-centered Values)**	-	Ethical and normative guidelines	Multi-stakeholder and adaptive governance and collaboration	-	-

**Table 3:** Selected criteria from the identified requirements for high-risk AI systems and qualitative methods aiming to assess those criteria (derived from the SCS ([23]) and extended by the authors).

Requirement	New ID	SCS ID	SCS Aspects as Discussed in Holzinger et al. (2020) ([23] or New Criteria
**Risk management**	$RM_{i}$		
**Cybersecurity**	$CS_{i}$		
**Data Protection (incl. Privacy Protection)**	$DP_{i}$		
**Reliability and Safety and Run-time Constraints**	$RRT_{1}$	10	I received the explanations in a timely and efficient manner.
**Accountability, Autonomy and Control**	$AAC_{1}$	2	I understood the explanations within the context of my work.
	$AAC_{2}$	3	I could change the level of detail on demand.
	$AAC_{3}$	4	I did not need support to understand the explanations.
	$AAC_{4}$	5	I found the explanations helped me to understand causality.
	$AAC_{5}$	7	I did not find inconsistencies between explanations.
	$AAC_{6}$	10	I received the explanations in a timely and efficient manner.
**Transparency of Algorithms**	$TA_{1}$	new	Is the algorithm published?
	$TA_{2}$	new	Is the algorithm accepted by the corresponding community?
**Algorithm Auditability and Explainability**	$AAE_{1}$	4	I did not need support to understand the explanations.
**Usability, User Interface Design, and Fairness (accessibility)**	$UIF_{1}$	2	I understood the explanations within the context of my work.
	$UIF_{2}$	3	I could change the level of detail on demand.
	$UIF_{3}$	4	I did not need support to understand the explanations.
	$UIF_{4}$	8	I think that most people would learn to understand the explanations very quickly.
	$UIF_{5}$	9	I did not need more references in the explanations: e.g., medical guidelines, regulations.
**Accuracy, Decision Confidence, and Reproduceability**	$ADC_{1}$	new	I repeated the processing on the same data and came to the same results and conclusions.
**Fairness** (non-biased decisisons)	$FNB_{i}$		
**Decision Interpretability and Explainability** (operator’s perspective)	$DIE_{1}$	1	I found that the data included all relevant known causal factors with sufficient precision and granularity.
	$DIE_{2}$	2	I understood the explanations within the context of my work.
	$DIE_{3}$	3	I could change the level of detail on demand.
	$DIE_{4}$	4	I did not need support to understand the explanations.
	$DIE_{5}$	5	I found the explanations helped me to understand causality.
	$DIE_{6}$	6	I was able to use the explanations with my knowledge base.
	$DIE_{7}$	7	I did not find inconsistencies between explanations.
	$DIE_{8}$	8	I think that most people would learn to understand the explanations very quickly.
	$DIE_{9}$	10	I received the explanations in a timely and efficient manner.
	$DIE_{10}$	new	I would be able to explain the decision (and its reason(s)) to another operator.
	$DIE_{11}$	new	I would be able to explain the decision (and its reason(s)) to an affected entity.
**Transparency and Contestability of Decisions** (affected entity/-ies’ perspective)	$TCD_{1}$	1	I found that the data included all relevant known causal factors with sufficient precision and granularity.
	$TCD_{2}$	3	I could change the level of detail on demand.
	$TCD_{3}$	4	I did not need support to understand the explanations.
	$TCD_{4}$	5	I found the explanations helped me to understand causality
	$TCD_{5}$	8	I think that most people would learn to understand the explanations very quickly.

## Data Availability

No new data were created.

## References

[1] European Commission (2021). Proposal for a Regulation of the European Parliament and of the Council Laying Down Harmonised Rules on Artificial Intelligence (Artificial Intelligence Act) and Amending Certain Union Legislative Acts. COM(2021) 206 Final. https://eur-lex.europa.eu/legal-content/EN/TXT/?uri=CELEX:52021PC0206.

[2] European Parliament (2023). Amendments Adopted by the European Parliament on 14 June 2023 on the Proposal for a Regulation of the European Parliament and of the Council on Laying Down Harmonised Rules on Artificial Intelligence (Artificial Intelligence Act) and Amending Certain Union Legislative Acts. COM(2021)0206–C9-0146/2021–2021/0106(COD). https://www.europarl.europa.eu/doceo/document/TA-9-2023-0236_EN.html.

[3] Rathgeb C., Tolosana R., Vera-Rodriguez R., Busch C. (2022). Handbook of Digital Face Manipulation and Detection From DeepFakes to Morphing Attacks.

[4] Siegel D., Krätzer C., Seidlitz S., Dittmann J. (2021). Media Forensics Considerations on DeepFake Detection with Hand-Crafted Features. J. Imaging.

[5] Siegel D., Krätzer C., Seidlitz S., Dittmann J. (2022). Forensic data model for artificial intelligence based media forensics - Illustrated on the example of DeepFake detection. Electron. Imaging.

[6] U.S. Congress (2021). Federal Rules of Evidence; Amended by the United States Supreme Court in 2021.

[7] Legal Information Institute (2019). Rule 702. Testimony by Expert Witnesses. https://www.law.cornell.edu/rules/fre/rule_702.

[8] Champod C., Vuille J. (2011). Scientific Evidence in Europe–Admissibility, Evaluation and Equality of Arms. Int. Comment. Evid..

[9] BSI (2011). Leitfaden IT-Forensik.

[10] Kiltz S. (2020). Data-Centric Examination Approach (DCEA) for a Qualitative Determination of Error, Loss and Uncertainty in Digital and Digitised Forensics. Ph.D. Thesis.

[11] European Network of Forensic Science Institutes (2021). Best Practice Manual for Digital Image Authentication. ENFSI-BPM-DI-03. https://enfsi.eu/wp-content/uploads/2022/12/1.-BPM_Image-Authentication_ENFSI-BPM-DI-03-1.pdf.

[12] Siegel D., Kraetzer C., Dittmann J. (2023). Joining of Data-driven Forensics and Multimedia Forensics for Deepfake Detection on the Example of Image and Video Data. Proceedings of the SECURWARE 2023, The Seventeenth International Conference on Emerging Security Information, Systems and Technologies.

[13] European Union Agency For Cybersecurity (2022). Remote Identity Proofing: Attacks & Countermeasures. Technical Report. https://www.enisa.europa.eu/publications/remote-identity-proofing-attacks-countermeasures.

[14] European Commission (2020). Assessment List for Trustworthy Artificial Intelligence (ALTAI) for Self-Assessment. https://digital-strategy.ec.europa.eu/en/library/assessment-list-trustworthy-artificial-intelligence-altai-self-assessment.

[15] Wing J.M. (2021). Trustworthy AI. Commun. ACM.

[16] Phillips P.J., Hahn C.A., Fontana P.C., Yates A.N., Greene K., Broniatowski D.A., Przybocki M.A. (2021). Four principles of Explainable Artificial Intelligence.

[17] UNICRI, INTERPOL (2023). Toolkit for Responsible AI Innovation in Law Enforcement: Principles for Responsible AI Innovation. Technical Report. https://unicri.it/sites/default/files/2023-06/02_Principles%20for%20Responding%20AI%20Innovation.pdf.

[18] Wahlster W., Winterhalter C. (2020). German Standardization Roadmap on Artificial Intelligence.

[19] Berghoff C., Biggio B., Brummel E., Danos V., Doms T., Ehrich H., Gantevoort T., Hammer B., Iden J., Jacob S. (2021). Towards Auditable AI Systems–Current Status and Future Directions.

[20] European Commission (2016). Regulation (EU) 2016/679 of the European Parliament and of the Council of 27 April 2016 on the Protection of Natural Persons with Regard to the Processing of Personal Data and on the Free Movement of such Data, and Repealing Directive 95/46/EC (General Data Protection Regulation). https://eur-lex.europa.eu/legal-content/EN/TXT/?uri=CELEX:02016R0679-20160504.

[21] Kraetzer C., Siegel D., Seidlitz S., Dittmann J. (2022). Process-Driven Modelling of Media Forensic Investigations-Considerations on the Example of DeepFake Detection. Sensors.

[22] Linardatos P., Papastefanopoulos V., Kotsiantis S. (2021). Explainable AI: A Review of Machine Learning Interpretability Methods. Entropy.

[23] Holzinger A., Carrington A., Müller H. (2020). Measuring the Quality of Explanations: The System Causability Scale (SCS). KI-Künstliche Intell..

[24] Likert R. (1932). A technique for the measurement of attitudes/by Rensis Likert. Arch. Psychol..

[25] Rosenfeld A., Dignum F., Lomuscio A., Endriss U., Nowé A. (2021). Better Metrics for Evaluating Explainable Artificial Intelligence. Proceedings of the AAMAS ’21: 20th International Conference on Autonomous Agents and Multiagent Systems.

[26] Jüttner V., Grimmer M., Buchmann E. (2023). ChatIDS: Explainable Cybersecurity Using Generative AI. Proceedings of the SECURWARE 2023, The Seventeenth International Conference on Emerging Security Information, Systems and Technologies.

[27] Lapuschkin S., Binder A., Montavon G., Müller K.R., Samek W. (2016). The LRP Toolbox for Artificial Neural Networks. J. Mach. Learn. Res..

[28] Ribeiro M.T., Singh S., Guestrin C. “Why Should I Trust You?”: Explaining the Predictions of Any Classifier. Proceedings of the 22nd ACM SIGKDD International Conference on Knowledge Discovery and Data Mining.

[29] Lundberg S.M., Lee S.I., Guyon I., Luxburg U.V., Bengio S., Wallach H., Fergus R., Vishwanathan S., Garnett R. (2017). A Unified Approach to Interpreting Model Predictions. Advances in Neural Information Processing Systems.

[30] Chattopadhyay A., Sarkar A., Howlader P., Balasubramanian V.N. (2018). Grad-CAM++: Generalized Gradient-Based Visual Explanations for Deep Convolutional Networks. Proceedings of the 2018 IEEE Winter Conference on Applications of Computer Vision, WACV 2018.

[31] Gossen F., Margaria T., Steffen B. (2020). Towards Explainability in Machine Learning: The Formal Methods Way. IT Prof..

[32] Rudin C. (2019). Stop explaining black box machine learning models for high stakes decisions and use interpretable models instead. Nat. Mach. Intell..

[33] European Commission (2019). Communication from the Commission to the European Parliament, the Council, the European Economic and Social Committee and the Regions:Building Trust in Human-Centric Artificial Intelligence. COM(2019) 168 Final. https://eur-lex.europa.eu/legal-content/EN/TXT/?uri=CELEX%3A52019DC0168&qid=1707400044663.

[34] European Commission (2019). Independent High-Level Expert Group on Artificial Intelligence set up by the European Commision Ethics Guidlines for Trustworthy AI. https://ec.europa.eu/newsroom/dae/redirection/document/60419.

[35] Krätzer C., Siegel D., Seidlitz S., Dittmann J. (2023). Human-in-control and quality assurance aspects for a benchmarking framework for DeepFake detection models. Electron. Imaging.

[36] Unesco Draft Text of the Recommendation on the Ethics of Artifical Intelligence. In Proceedings of the Intergovernmental Meeting of Experts (Category II) Related to a Draft Recommendation on the Ethics of Artificial Intelligence, Online, 21–25 June 2021. https://unesdoc.unesco.org/ark:/48223/pf0000377897.

[37] Roccetti M., Delnevo G., Casini L., Salomoni P. (2020). A Cautionary Tale for Machine Learning Design: Why we Still Need Human-Assisted Big Data Analysis. Mob. Networks Appl..

[38] Cui Y., Koppol P., Admoni H., Niekum S., Simmons R., Steinfeld A., Fitzgerald T., Zhou Z.H. (2021). Understanding the Relationship between Interactions and Outcomes in Human-in-the-Loop Machine Learning. Proceedings of the Thirtieth International Joint Conference on Artificial Intelligence, IJCAI-21.

[39] Jena S., Sundarrajan S., Meena A., Chandavarkar B.R., Misra R., Kesswani N., Rajarajan M., Veeravalli B., Brigui I., Patel A., Singh T.N. (2023). Human-in-the-Loop Control and Security for Intelligent Cyber-Physical Systems (CPSs) and IoT. Proceedings of the Advances in Data Science and Artificial Intelligence.

[40] Chen C., Li O., Tao D., Barnett A., Rudin C., Su J., Wallach H.M., Larochelle H., Beygelzimer A., d’Alché-Buc F., Fox E.B., Garnett R. (2019). This Looks Like That: Deep Learning for Interpretable Image Recognition. Proceedings of the Advances in Neural Information Processing Systems 32: Annual Conference on Neural Information Processing Systems 2019, NeurIPS 2019.

[41] Leventi-Peetz A., Östreich T. (2022). Deep Learning Reproducibility and Explainable AI (XAI).

[42] Liao Q.V., Gruen D., Miller S. (2020). Questioning the AI: Informing Design Practices for Explainable AI User Experiences. Proceedings of the 2020 CHI Conference on Human Factors in Computing Systems.

